# Genomic landscape of pancreatic neuroendocrine tumours: the International Cancer Genome Consortium

**DOI:** 10.1530/JOE-17-0560

**Published:** 2018-01-10

**Authors:** Andrea Mafficini, Aldo Scarpa

**Affiliations:** 1 ARC-Net Centre for Applied Research on CancerUniversity and Hospital Trust of Verona, Verona, Italy; 2 Department of Diagnostics and Public HealthSection of Pathology, University and Hospital Trust of Verona, Verona, Italy

**Keywords:** neuroendocrinology, pancreas, cancer, molecular biology

## Abstract

Neuroendocrine tumours (NETs) may arise throughout the body and are a highly heterogeneous, relatively rare class of neoplasms difficult to study also for the lack of disease models. Despite this, knowledge on their molecular alterations has expanded in the latest years, also building from genetic syndromes causing their onset. Pancreatic NETs (PanNETs) have been among the most studied, and research so far has outlined a series of recurring features, as inactivation of *MEN1*, *VHL*, *TSC1/2* genes and hyperactivation of the PI3K/mTOR pathway. Next-generation sequencing has added new information by showing the key role of alternative lengthening of telomeres, driven in a fraction of PanNETs by inactivation of *ATRX*/*DAXX.* Despite this accumulation of knowledge, single studies often relied on few cases or were limited to the DNA, RNA, protein or epigenetic level with lack of integrative analysis. The International Cancer Genome Consortium aimed at removing these barriers through a strict process of data and samples collection, to produce whole-genome integrated analyses for many tumour types. The results of this effort on PanNETs have been recently published and, while confirming previous observations provide a first snapshot of how heterogeneous is the combination of genetic alterations that drive this tumour type, yet converging into four pathways whose alteration has been enriched by newly discovered mechanisms. While calling for further integration of genetic and epigenetic analyses, these data allow to reconcile previous findings in a defined frame and may provide clinical research with markers for patients stratification and to guide targeted therapy decisions.

## Introduction

Neuroendocrine tumours (NETs) are relatively rare, with an incidence of 3–5 cases per 100,000 individuals per year in the United States and Europe, and have a variable behaviour from relatively indolent to highly aggressive (Lloyd *et al*. 2017). However, a large number of these tumours have a prolonged clinical course, this causing their prevalence to be one of the highest, second only to colorectal cancer according to the National Cancer Institute Surveillance, Epidemiology, and End Results program (Yao *et al*. 2008, Dasari *et al*. 2017). This observation, together with the fact that the incidence of these tumours has been steadily increasing in the last 30 years, has risen the interest on a better understanding of this largely heterogeneous group of tumours to guide a better management of patients.

NETs may arise in different sites, and the largest group is constituted by gastroenteropancreatic NETs (GEP-NETs, 61%). Despite having been initially known as cause of the ‘carcinoid syndrome’, most GEP-NETs may be asymptomatic for a long time or present with non-specific symptoms. As a result, a large fraction of patients are diagnosed with advanced or metastatic disease, and the mortality rate is 50% (Scherubl *et al*. 2013). Due to the lack of disease models, that would be anyhow limited by the large heterogeneity of these tumours, most of the research on their biology has been undertaken by investigating tumour specimens (Capdevila *et al*. 2017). A small fraction (10%) of NETs arise in the context of familial syndromes, and the genetic alterations linked to those syndromes constituted the first information on the biology of NETs that was also investigated in sporadic tumours. The advent of powerful high-throughput techniques such as next-generation sequencing allowed a deeper, unbiased exploration of tumour specimens, which led to the progressive accumulation of further knowledge on the mechanisms that drive the progression towards malignancy (Scarpa *et al*. 2015). However, these studies still lacked the coordinated efforts to collect, select and analyse the whole genome of a large number of cases, which has been the flagship of the International Cancer Genome Consortium (ICGC) (International Cancer Genome Consortium**2010).

In the ICGC framework, the Italian-Australian initiative has been involved in the analysis of pancreatic adenocarcinoma and rare pancreatic neoplasms. The latter section of the project recently led to publishing the results of whole-genome analysis on 98 pancreatic NETs (PanNETs). This study, while confirming the previous knowledge, adds interesting information and a broader understanding of how mutations, chromosomal alterations and gene expression converge on specific pathways (PI3K/mTOR, DNA damage repair, chromatin modification) more often than previously expected, and with new players (Scarpa *et al*. 2017). In this focused review, we will summarise our knowledge on PanNETs before (Table 1) the publication of the ICGC group results and will then discuss the contribution of the latter to the building of a more complete picture of the molecular biology of PanNETs. Finally, we will give an overview of the open questions and expected developments after this milestone, especially with regard to targeted therapy (Capdevila *et al*. 2017, Gajate *et al*. 2017).

**Table 1:** Genes implicated in the tumorigenesis of pancreatic neuroendocrine tumours (PanNETs) before the publication of the International Cancer Genome Consortium data.

Gene/locus	First information source	Pathway
*MEN1*	Genetic syndrome (multiple endocrine neoplasia type I)	Chromatin remodeling, altered telomeres, PI3K/mTOR DNA double-strand break repair
*VHL*	Genetic syndrome (Von-Hippel Lindau)	PI3K/mTOR
*NF1*	Genetic syndrome (neurofibromatosis type I)	PI3K/mTOR, Ras/MAPK
*TSC1/TSC2*	Genetic syndrome (tuberous sclerosis)	PI3K/mTOR
*PTEN*	Next-generation sequencing of PanNETs	PI3K/mTOR
*ATRX*	Next-generation sequencing of PanNETs	Telomeres elongation
*DAXX*	Next-generation sequencing of PanNETs	Telomeres elongation, PI3K/mTOR
1q, 3p, 6q, 10q, 11q	Chromosomal abherrations of PanNETs (LOSS)	–
7p, 9p, 17q, 20q	Chromosomal abherrations of PanNETs (GAIN)	–
*RASSF1A*	Promoter hypermethylation in PanNETs	Ras/MAPK
*CDKN2A*	Promoter hypermethylation in PanNETs	Cell cycle
*ALU*, *LINE1*	Promoter hypomethylation in PanNETs	–
*miR-21*	miRNA differential expression in PanNETs	PI3K/mTOR

## The molecular picture of PanNETs from hereditary syndromes and pre-ICGC studies

Inherited cancer predisposition syndromes account for about one-tenth of diagnosed NETs. The genetic study of syndromic subjects and their families provided the first knowledge of pivotal genes that, when altered in the germline, predispose to endocrine tumour onset. The *MEN1* gene, identified in patients suffering from multiple endocrine neoplasia type I, has been proven to be altered also in a large fraction (44%) of sporadic PanNETs (Corbo *et al*. 2010, Jiao *et al*. 2011) and in a smaller but significant group of pulmonary carcinoids, where it was linked to poorer prognosis (Swarts *et al*. 2014). The gene product, menin, has several activities linked to its ability to modulate histones methylation, that may be summarised in three broader functions: (i) negative regulation of the cell cycle via increased expression of *CDKN2C*/*CDKN1B*, (ii) inhibition of the PI3K/mTOR signalling pathway via regulation of AKT1 cellular localisation and (iii) promotion of DNA double-strand breaks repair through activation of genes belonging to the homologous recombination (HR) DNA repair machinery like *BRCA1* and *RAD51*. The PI3K/mTOR pathway is also affected by three other genetic syndromes that may result in NETs development: Von-Hippel-Lindau (VHL) disease, neurofibromatosis type I (NF1) and tuberous sclerosis (TS). VHL is caused by inactivating mutations in the namesake gene, which is a negative regulator of the of the hypoxia-induced pro-proliferative *HIF1* gene, a downstream effector of the PI3K/mTOR pathway as well; the *VHL* gene has also been reported to be frequently inactivated by non-mutational mechanisms in up to 25% of sporadic PanNETs (Scarpa *et al*. 2015). Germline mutation of the *NF1* gene is the cause of neurofibromatosis type I, as the gene product neurofibromin is a negative regulator of the Ras/MAPK and PI3K/mTOR signal transduction networks; its involvement in sporadic PanNETs is occasional, while about 40% of periampullary duodenal somatostatinomas associate with NF1 disease. Finally, TS arises by inactivating mutation of either of two genes (*TSC1* and *TSC2*), which act as a complex to inhibit PI3K/mTOR signalling downstream of AKT1. While TS rarely results in PanNETs, recent works have shown that *TSC2* downregulation and mutation affect around 35% and 9% of sporadic PanNETs, respectively (Missiaglia *et al*. 2010, Jiao *et al*. 2011). The latter data about *TSC2* mutation in sporadic PanNET was produced by the first whole-exome study of this neoplasia, including 68 cases (Jiao *et al*. 2011). This study also confirmed previous low-throughput studies on the frequent mutation of *MEN1*, and identified *PTEN*, another gene of the PI3K/mTOR pathway involved in the inhibition of PI3K, as a recurrently (7.3%) mutated tumour suppressor.

Moreover, two novel genes were found frequently mutated that flanked *MEN1* in the category of the chromatin remodeling genes implicated in PanNET carcinogenesis: *ATRX* and *DAXX* (Jiao *et al*. 2011). These genes were affected by inactivating mutation in 18% and 25% of PanNETs, with mutations in each other gene showing no overlap. This mutual exclusivity of *ATRX*/*DAXX* mutations led to the hypothesis of a physical interaction between the two gene products, that was confirmed by subsequent research; indeed both DAXX and ATRX interact to bind and deposit histone H3.3 on several sites, including the centromere and the telomeres (Lewis *et al*. 2010). The inactivation of this complex has been linked to chromosomal instability, poorer prognosis and to a phenomenon called alternative lengthening of telomeres (ALT), where telomeres are elongated in a telomerase-independent way. This association has been described specifically in PanNETs and in correlation with higher stage tumours, suggesting that *ATRX/DAXX* inactivation is a late event of the neoplastic transformation (Heaphy *et al*. 2011, de Wilde *et al*. 2012, Marinoni *et al*. 2014). Mutations in chromatin remodeling (i.e. *MEN1*, *ATRX*, *DAXX*) and HR DNA repair genes either directly involved in DNA repair (*BRCA2)* or activated upon double-strand break detection and controlling the intersection between cell cycle, DNA repair and apoptosis (*CHEK2*), support the idea of a lead role for chromosomal and epigenetic alterations in the tumorigenesis of PanNET. Indeed, mutations alone provide a rationale for tumorigenesis only in about 40% of these neoplasms. This means that in the remaining cases, the initiation of the neoplastic process may lean on chromosomal/epigenetic alterations rather than on a recurrent driver mutation. Notably, chromatin remodeling and DNA repair genes, albeit different from those specific for PanNETs, have been implicated also in lung NETs by recent genomic studies (Fernandez-Cuesta *et al*. 2014, Simbolo *et al*. 2017).

As for chromosomal alterations, several studies have been published and showed a high degree of aberrations in PanNETs; these included frequent loss of chromosome 1q, 3p (where the *VHL* gene is located) and 11q (where *MEN1* and also *ATM*, another pivotal gene activated by DNA double-strand breaks and involved in both damage checkpoint and double-strand repair, reside). Other losses were frequent but showed less concordance among different studies, including chromosome 6q, 10q (where *PTEN* resides) and 11p. Tumours also showed recurrent gains at chromosome 7q and 9q, while variable gains were located at chromosome 7p, 9p, 17q and 20q, as reviewed in Capurso *et al*. (2012). Meanwhile, DNA methylation studies have shown frequent hyper-methylation in a large fraction of PanNETs and identified several target genes/promoters including *RASSF1A*, *CDKN2A* and *VHL*. Hypo-methylation was also reported for *ALU* and* LINE1*, the latter being correlated with poor prognosis and chromosomal instability of *ATRX*/*DAXX*-negative tumours (Stefanoli *et al*. 2014, Di Domenico *et al*. 2017, Marinoni *et al*. 2017). This combination of diverse genetic and epigenetic alterations may be puzzling to solve but could result in more homogeneous downstream effects; therefore, researches also tackled the gene expression profiling of PanNETs. Both miRNA and mRNA profiles of primary tumours were analysed by separate studies. The study of miRNA allowed to identify a set of miRNAs that distinguish neuroendocrine from acinar tumours, also revealing the association between overexpression of miR-21 (that sustains PI3K/mTOR pathways through inhibition of *PTEN*) and PanNETs with high proliferation index and liver metastasis (Roldo *et al*. 2006, Karpathakis *et al*. 2013).

The PI3K/mTOR pathway was again implicated when analysing the expression profiles of PanNETs, with a particularly evident association between low *PTEN* and *TSC2* expression levels and development of metastasis, tumour progression and poor overall survival (Missiaglia *et al*. 2010). This study also showed the *in vitro* efficacy of mTOR inhibition using PanNET cell lines that displayed reduced levels of *PTEN* and *TSC2*. More recently, an integrated study was performed in the attempt to subgroup PanNETs by simultaneous comparison of miRNA, mRNA and mutation profiles and by cross-comparing human tumours with those arising in the Rip1Tag2 mouse model of pancreas neuroendocrine tumours (Sadanandam *et al*. 2015). Three subtypes of tumour were identified by miRNA/mRNA profiles of both human and murine specimens: insulinoma-like tumours (IT), intermediate MEN1-like tumours and metastasis-like primary tumours (MLP). However, while IT and MLP tumours from human and mouse clustered together, MEN1-like tumours formed a compact subgroup that clustered far from the mouse model and were enriched in mutations of chromatin remodeling genes (*MEN1*, *DAXX*, *ATRX*). By contrast, IT tumours were enriched in mutations affecting the PI3K/mTOR pathway (*TSC2*, *PTEN*), while in the MLP subtype, both chromatin remodeling and PI3K/mTOR were affected (Sadanandam *et al*. 2015). Each of the previous studies, even those with the larger throughput and number of cases, could provide only a partial view of the complex molecular dysregulation that drivers PanNET oncogenesis. Furthermore, despite the apparent implication of the PI3K/mTOR pathways in at least a subset of PanNETs, a rationale for stratification of patients that could benefit from everolimus or other mTOR inhibitors could not be reached, and the hyperactivation of the mTORC2 complex in reaction to mTORC1 inhibition (Kim *et al*. 2017) in PI3K/mTOR dysregulated PanNETs has not been fully dissected yet. The ICGC consortium was specifically born to coordinate large worldwide studies that could collect and process high quality homogeneous data from whole genome, exome, transcriptome, microarray genomic hybridisation and ancillary techniques, on a large number of carefully verified tumour samples for each cancer type to be profiled (International Cancer Genome Consortium**2010). The data produced from each project not only can confirm or confute previous data or produce new insights, but also can offer a scaffold to recompose sparse reports as in the case of rare neoplasms.

## The genomic landscape of PanNETs according to the ICGC data

The ICGC whole-genome study was aimed at studying well-differentiated PanNETs, mostly of G1–G2 grade, and used 160 cases (98 in the discovery cohort and 62 in the validation cohort), excluding poorly differentiated pancreatic neuroendocrine carcinomas, mixed adenoneuroendocrine carcinomas and familial cases. Mutational analysis and comparison with previous data on pancreatic ductal adenocarcinoma (Waddell *et al*. 2015) confirmed the low mutational load of PanNETs. The first novel finding of the ICGC study is the definition of mutational signatures that characterise these tumours, which includes 4 processes already described in other tumours (APOBEC, age, BRCA and the unknown aetiology ‘Signature 5’) (Alexandrov *et al*. 2013) and a previously unreported base excision-repair (BER) deficiency signature due to the biallelic inactivation of the *MUTYH* gene due to pathogenic germline mutations and somatic loss of heterozygosity; this signature has been shown therein to be the same of MUTYH-associated polyposis (MAP) of the colon (Scarpa *et al*. 2017), and this finding has been confirmed by a recent publication that also reported the same signature in a subset of adrenocortical carcinomas (Pilati *et al*. 2017). The second novel finding relies in the higher than expected prevalence of germline mutations, which were found in 17% of patients lacking a family or personal history of cancer and affected the known *MEN1*, *VHL* and *CDKN1B* genes, and the previously unreported DNA damage repair genes *MUTYH*, *CHEK2* and *BRCA2*. The third novel finding is the identification of novel mutational mechanisms including a pattern of chromosomal rearrangements compatible with chromothripsis in 9% of cases and *EWSR1* gene fusions (known as a driver alteration in Ewing’s sarcoma) in 3% of tumours. The fourth relevant observation regards the analysis of somatically altered driver genes that confirmed a heavy involvement of the chromatin remodeling and PI3K/mTOR pathway in PanNETs development, with *MEN1* (36 cases) as the top driver gene, *DAXX* (22 cases) and *ATRX* (10 mutated, one rearranged case) mutually exclusive alterations as the second main event and linked to ALT, followed by mutations in members of the PI3K/mTOR pathway as *PTEN* (7 cases), *TSC1/2* (3 cases) and the previously undetected *DEPDC5* (2 cases), which is part of the GATOR1 complex inhibiting mTORC1 activator Rag (Bar-Peled *et al*. 2013). The inactivation of *SETD2*, a histone modifier tumour suppressor gene (Li *et al*. 2016) in 6% of cases (mutation in 5 cases and rearrangement in one case) is another previously unreported recurrent alteration in PanNETs. Other chromatin remodeling genes previously reported as inactivated in lung NETs (Fernandez-Cuesta *et al*. 2014, Simbolo *et al*. 2017) were inactivated by structural rearrangements, namely *ARID2* (5 cases), *SMARCA4* (3 cases) and *KMT2C/MLL3* (3 cases). The PI3K/mTOR pathway was involved by three novel findings: the rearrangement leading to fusion transcripts of the *EWSR1* gene (3 cases), a possible marker for PI3K/mTOR targeted therapy (Giorgi *et al*. 2015) and the recurrent amplification of PI3K’s activator *PSPN* and mTOR’s regulator *ULK1*. The fifth relevant finding relates to RNAseq clustering of 30 cases, which confirmed the 3 subgroups previously described (Sadanandam *et al*. 2015) showing a prominent involvement of hypoxia-related genes in the MLP subgroup.

**Figure 1:**
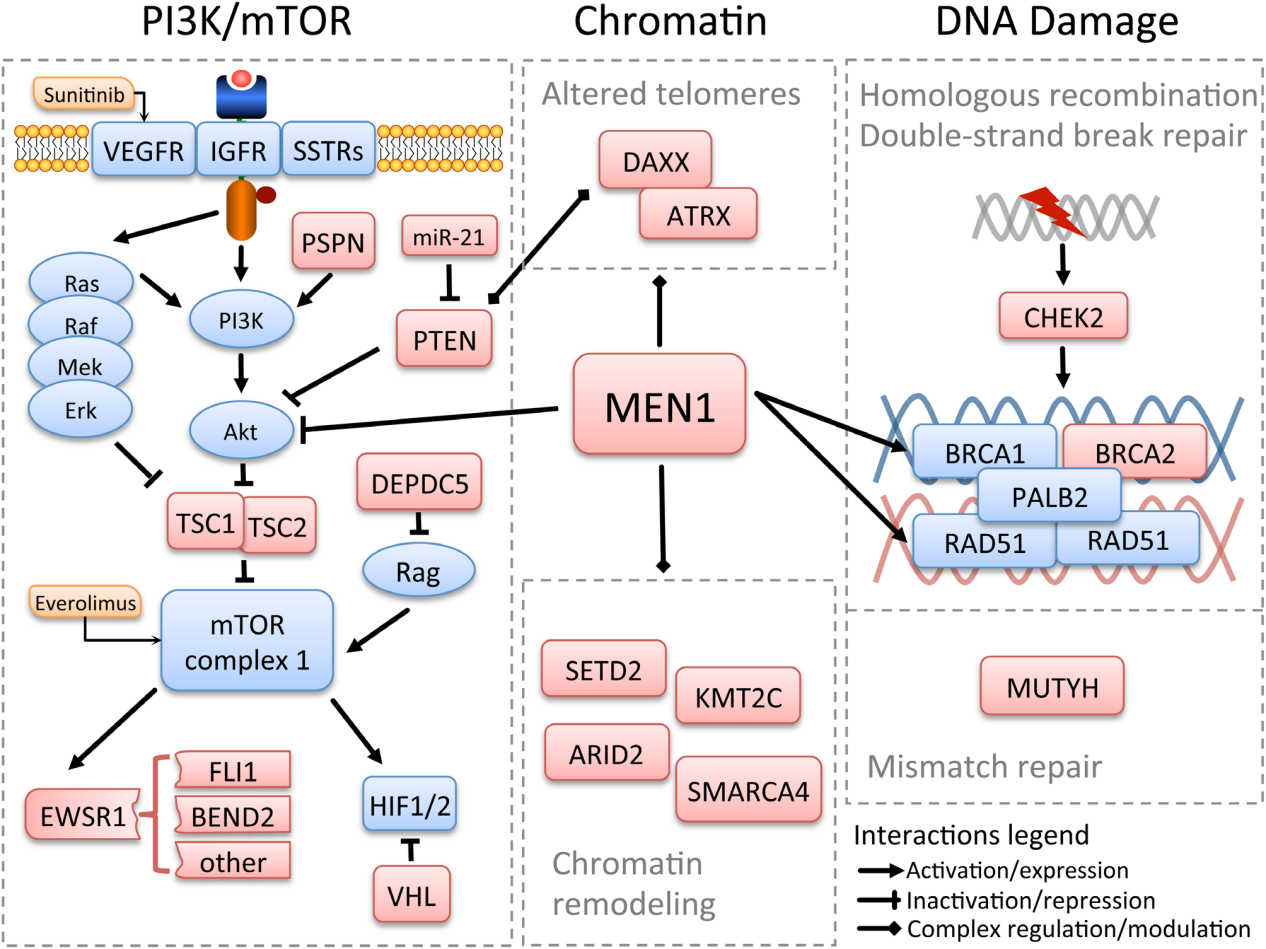
Outline of main altered pathways in pancreatic neuroendocrine tumours. The scheme merges data from the literature and published data of the International Cancer Genome Consortium (ICGC). Pathway members whose genetic alteration has been proven are shaded in red, approved targeted drugs are shaded in orange. *MEN1* interacts and modulates all core pathways acting as a hub gene. DAXX/ATRX also cooperate with the other genes of the chromatin remodeling complexes. DAXX modulates PTEN distribution between the nucleus and the cytoplasm (Song *et al*. 2008), and the modulation of *DAXX* by PTEN has been reported soon after publication of the ICGC data (Benitez *et al*. 2017).

Four core pathways (Fig. 1) of PanNETs dominated by the presence of altered *MEN1*, outlined by previous research, emerge from this report with a better definition of genes involved as players, including DNA damage repair, chromatin remodeling, telomere alteration and the PI3K/mTOR signalling pathway. DNA damage repair was altered by germline mutations more often than expected, opening a question on the actual heritability level in these tumours; moreover, frequent alterations of the HR complex open the door to evaluate the applicability of therapeutic strategies (platinum or PARP-inhibitors) already approved for other HR-deficient cancers. Chromatin remodeling alterations involved not only *MEN1*, that was however the dominant driver in most cases, but also other complexes such as the SWI/SNF and the histone methylase complex whose alterations PanNETs share with lung NETs. This may lead to postulate a common ground for chromatin reprogramming in NETs of different districts, also opening a chapter of PanNETs research that deserves further studies to bridge genetic and epigenetic alterations. Telomere alteration by *DAXX*/*ATRX* inactivation was confirmed, while the effect of *MEN1* alteration showed a more variable effect, compatible with the many functions of menin itself. *DAXX*/*ATRX* mutations also confirmed their value as predictor of poor prognosis: subjects bearing tumours with inactive *DAXX/ATRX* had a lower disease-specific survival, as also outlined by another recent study on 321 PanNET patients that reported an association between ALT-positive, DAXX/ATRX-negative PanNETs and shorter disease-free survival (Singhi *et al*. 2017). The landscape of PI3K/mTOR pathway alterations was enriched by new potential players, like *DEPDC5* and *EWSR1* fusions, and correlated with both the presence of *DAXX*/*ATRX* mutation and poor prognosis, a further aid for patients stratification that should be validated with larger cohorts to define the best combination of alterations with the highest independent predictive value. Moreover, the novel information on the dysregulation of the PI3K/mTOR pathway may be used as a source for biomarkers that should be tested in selected patients (e.g. those with *EWSR1* fusion genes) as predictors of response in current and future clinical trials with drugs targeting this pathway.

## Conclusion

The ICGC data on PanNETs allowed to draw the first snapshot of the heterogeneous combination of genetic alterations that drive this type of tumours. Despite this variability, some key pathways are consistently targeted by the tumorigenic process, resulting in a frequent loss of *MEN1* function, activation of the PI3K/mTOR pathway, chromatin remodeling and telomeres alteration. While the next step for translational research will be the integration of genomic, transcriptomic and epigenomic data to identify key bottlenecks of this network and to explain how different alterations lead to a small number of subgroups according to expression profiles, these data may propel clinical research aimed at identifying and validating efficient markers for patients stratifications and to guide the choice of targeted therapeutic options, as recently outlined (Capdevila *et al*. 2017).

## Declaration of interest

The authors declare that there is no conflict of interest that could be perceived as prejudicing the impartiality of this review.

## Funding

This work was supported in part by Associazione Italiana per la Ricerca sul Cancro (grant n.12182); European Commission Seventh Framework Programme FP7 Health (Cam-Pac, grant agreement 602783).
